# Cognitive and Executive Function Scores at Age 7 in Relation to Maternal Mid-Pregnancy Plasma Nutrient Mixtures in a Singaporean Family Follow-Up Cohort

**DOI:** 10.3390/nu18050818

**Published:** 2026-03-03

**Authors:** Jordana Leader, Shiwen Li, Stefano Renzetti, Jun Shi Lai, Yap-Seng Chong, Kok Hian Tan, Johan G. Eriksson, Keith M. Godfrey, Evelyn C. Law, Mary Foong-Fong Chong, Shiao-Yng Chan, Damaskini Valvi, Jonathan Huang, Youssef Oulhote

**Affiliations:** 1 Department of Environmental Medicine, Icahn School of Medicine at Mount Sinai, New York, NY 10029, USA; dania.valvi@mssm.edu (D.V.); youssef.oulhote@mssm.edu (Y.O.); 2 Thompson School of Social Work and Public Health, Office of Public Health Studies, University of Hawaii at Manoa, Honolulu, HI 96822, USA; shiwenli@hawaii.edu (S.L.); huangjy@hawaii.edu (J.H.); 3 Department of Medical and Surgical Specialties, Radiological Sciences and Public Health, University of Brescia, 25123 Brescia, Italy; stefano.renzetti88@gmail.com; 4 Institute for Human Development and Potential, Agency for Science, Technology and Research, Singapore 117609, Singapore; junshilai@gmail.com (J.S.L.); paelecn@nus.edu.sg (E.C.L.); 5 Department of Obstetrics and Gynaecology, Yong Loo Lin School of Medicine, National University of Singapore, Singapore 117597, Singapore; obgcys@nus.edu.sg (Y.-S.C.); obgjge@nus.edu.sg (J.G.E.); obgchan@nus.edu.sg (S.-Y.C.); 6 Department of Obstetrics and Gynaecology, KK Women’s and Children’s Hospital, Singapore 229899, Singapore; gmstankh@nus.edu.sg; 7 MRC Lifecourse Epidemiology Centre, University of Southampton, Southampton SO16 6YD, UK; kmg@mrc.soton.ac.uk; 8 NIHR Southampton Biomedical Research Centre, University Hospital Southampton NHS Foundation Trust, Southampton SO16 6YD, UK; 9 Department of Paediatrics, Yong Loo Lin School of Medicine, National University of Singapore, Singapore 119228, Singapore; 10 Singapore Institute for Clinical Sciences, Agency for Science, Technology and Research, Singapore 117609, Singapore; 11 Saw Swee Hock School of Public Health, National University of Singapore, National University Health System, Singapore 117549, Singapore; mary_chong@nus.edu.sg

**Keywords:** prenatal nutrition, cognition, mixtures, child health, development

## Abstract

**Background**: Although there is substantial research into individual nutrients during pregnancy, such as folate, iron, and vitamin D, little is known about the impact of mixtures of essential nutrients. We investigated the associations between mixtures of maternal essential minerals and vitamin concentrations and child cognition and executive functions at age 7. **Methods**: Data from 348 mother–child pairs in the Growing up in Singapore Towards Healthy Outcomes birth cohort with both plasma nutrient and neurodevelopmental outcome data were used. Gestational fasting plasma samples between 26 and 28 weeks of gestation were analyzed for 10 essential minerals and 12 B and D vitamers. Child cognition and executive functions at 7 years were assessed using the Wechsler Abbreviated Scale of Intelligence 2nd Edition (WASI-II) [*n* = 331] and the Behavior Rating Inventory of Executive Function 2nd Edition (BRIEF-2) [*n* = 348], respectively. Generalized weighted quantile sum regression (gWQS) was used to investigate the associations between nutrient mixtures and child cognitive executive function scores. Single-nutrient analysis using covariate-adjusted multivariable regressions was performed as a sensitivity analysis. **Results**: A one-quartile increase in the positively weighted nutrient mixture index was associated with higher block design T-scores (β = 2.17, 95% CI: 0.03, 4.31). Additionally, the negatively weighted mixture was associated with lower block design (β = −2.25, 95% CI: −4.92, 0.41, *p* = 0.02) and perceptual reasoning (β = −1.94, 95% CI: −5.17, 1.29, *p* = 0.04) scores in boys only. We found no association between the nutrient mixture and BRIEF-2 subscale T-scores. **Conclusions**: In this study, we found that a positively weighted nutrient mixture index of maternal gestational minerals and vitamins was associated with a greater ability in children to analyze and understand abstract visual items.

## 1. Introduction

Nutrition during pregnancy impacts both the mother’s and the offspring’s health status. Overall, a more nutrient-rich diet has been found to be associated with better birth and child health outcomes [1,2]. Micronutrients are in higher demand during pregnancy due to increased maternal blood volume, metabolism, kidney function, and circulating hormones [3]. This often results in millions of pregnant women being deficient in nutrients such as iron, folate, and zinc [4]. It is for this reason that mothers are often encouraged by their physicians to take supplements during pregnancy.

Neurodevelopmental disorders affect approximately 8–34% of children and adolescents worldwide, with the prevalence in South-East Asia being 15% [5]. Brain development during pregnancy can impact cognitive, motor, and socio-emotional skills throughout life [6,7]. Nutrition during pregnancy is particularly crucial, as it is a sensitive period where multiple functions are developing, especially the central nervous system. More specifically, certain nutrients such as iron and folate have been identified as particularly beneficial to child neurodevelopment [3,8,9], while others have been shown to have detrimental effects in higher concentrations, e.g., manganese [10]. Additionally, other essential nutrients and vitamins such as copper, selenium, calcium, magnesium, vitamin D, and B12 have been linked to child cognition and executive function [3,7,9,11,12]. Although substantial research has been conducted on individual nutrients such as folate, iron, and vitamin D, there remains a gap in the literature examining the mixture of nutrients women consume either through supplementation or diet. Additionally, most of the previous studies investigated nutrient intake rather than objective measures of gestational nutrient concentrations. Through this research, we explored the associations between mixtures of maternal mineral and vitamin concentrations and child cognition and executive function, which can help influence future guidelines for prenatal nutrition.

## 2. Materials and Methods

### 2.1. Study Population

We included a subset of participants from the ongoing Growing up in Singapore Towards Healthy Outcomes (GUSTO) birth cohort study with available neurodevelopmental scores and nutrient concentrations. Details of the GUSTO cohort have been outlined in previous publications [13]. Briefly, the primary objective of the GUSTO study is to evaluate exposures and influences in early development on adverse metabolic functions and body composition later in life. The study enrolled 1468 pregnant women at least 18 years of age between June 2009 and September 2010 attending their first trimester antenatal dating ultrasound scan clinic at Singapore’s two major public maternity units. Chinese, Malay, or Indian participants were Singapore citizens or permanent residents. Women receiving chemotherapy, psychotropic drugs, or those with type I diabetes mellitus were excluded from the study. This study was approved by the National Health Care Group Domain Specific Review Board (DSRB D/2009/00021, approval date: 26 February 2009; DSRB B/2014/00414, approval date: 30 May 2014) and the SingHealth Centralized Institutional Review Board (CIRB 2018/2767/D, approval date: 2 March 2009). Written informed consent was obtained from all participants upon recruitment. Questionnaires were used to assess demographic, socioeconomic, lifestyle, maternal depression, anxiety, obstetric, and medical history data. Blood was collected at 26 to 28 weeks gestation for glucose tolerance testing and other biomarkers. Additionally, maternal and cord blood were collected at delivery to measure environmental chemicals.

### 2.2. Assessment of Maternal Nutrient Concentrations

Overnight fasting (8–10 h) blood samples were obtained from mothers at their 26–28 weeks’ gestation follow-up visit using standard venipuncture technique. The samples were processed and stored at −80 °C in EDTA tubes within 4 h of collection and thawed just prior to analysis. Maternal blood plasma samples were analyzed using inductively coupled plasma mass spectrometry (ICP-MS model NexIon 300D, PerkinElmer; Rotkreuz, Switzerland) for 10 minerals: sodium, magnesium, phosphorus, potassium, calcium, iron, ferritin, copper, zinc, and selenium. Twelve vitamins/vitamers: folate, B2-flavin, B2-neopterin, B2-riboflavin, B3-methylnicotinamide, B3-nicotinamide, B3-trigonelline, B6-pyridoxal, B6-pyridoxal 5-phosphate, B6-pyridoxic acid, B12, and D3 were measured using a targeted method based on liquid chromatography–tandem mass spectrometry (Bevital, Bergen, Norway) [14,15]. Additional analytical details for the process of the analysis of maternal plasma nutrient concentrations have been described in detail in other publications [16]. Nutrients were selected based on available measurements in the GUSTO biobank.

### 2.3. Child Cognition and Executive Function Assessment

Child cognition was measured using the Wechsler Abbreviated Scale of Intelligence 2nd Edition (WASI-II), administered by trained GUSTO staff at the year 7 childhood study visit. Child executive function was measured using the Behavior Rating Inventory of Executive Function 2nd Edition (BRIEF-2) questionnaire, completed by the child’s mother. The WASI-II is a brief, reliable measure of cognitive ability. It consists of 4 subtests: vocabulary and similarities, which are used to calculate the verbal intelligence index, and block design and matrix reasoning, which are used to calculate the perceptual reasoning index [17]. Higher WASI-II scale scores are associated with better cognition in that domain. For this analysis, we focused on block design, matrix reasoning, and perceptual reasoning index. The BRIEF-2 is a valid and reliable parent-reported questionnaire that measures the extent to which executive dysfunction impairs a child’s daily life. The BRIEF-2 includes 63 items with Likert-style responses that are used to calculate nine clinical scales, three broader indexes, and one composite scale [18]. Higher BRIEF-2 scores are associated with poorer executive function in that domain. For this analysis, we focused on the T-scores from three index scores: the behavior regulation index (BRI), the cognitive regulation index (CRI), the emotion regulation index (ERI), and the global executive composite (GEC) scale T-score.

### 2.4. Covariates

Covariates were selected as potential confounders or predictors of cognitive measures based on substantive a priori knowledge and data availability [19,20]. At the recruitment visit (<14 weeks’ gestation) and at 26–28 weeks’ gestation, questionnaires were administered to the women to capture demographic, socioeconomic, lifestyle, maternal well-being, obstetric, and medical history data. Additional questionnaires at delivery and during follow-up visits provided information about other characteristics. We considered the following covariates as potential confounders: child sex assigned at birth (male/female), child age at time of assessment, maternal age at recruitment (continuous), parity (nulliparous/primiparous/multiparous), maternal ethnicity (Chinese/Malay/Indian and other). We also considered maternal education, categorized as primary school or less, secondary school, vocational school or polytechnical school, and university or higher. Additionally, anthropometric measurements and BMI were calculated at 26–28 weeks’ gestation. Maternal BMI at 26 weeks was categorized as underweight (<18.5), normal weight (18.5–24.9), overweight (25–29.9), and obese (≥30).

### 2.5. Statistical Analysis

Means, standard deviations, and specific percentiles were used to summarize the distribution of cognitive measures, minerals, and vitamins/vitamers. We calculated descriptive statistics for participant demographics. Pearson’s correlation coefficients were used to assess the correlation between log 2 transformed nutrients. We also evaluated univariate associations between neurodevelopmental test scores and important characteristics using *t*-tests or analysis of variance, depending on the nature of the covariate. All analyses were performed using RStudio (version 4.5.2). 

#### 2.5.1. Mixtures Analysis

To investigate the potential joint effect of multiple nutrients, rather than individually, we conducted generalized weighted quantile sum regression (gWQS) [21]. gWQS regression is used to estimate the effects of multiple exposures and allows for possible different magnitudes and directions of associations for each group of chemicals. This allows the inclusion of two indices (one positive and one negative) in the same regression model [22]. We initially conducted an unadjusted gWQS with all 22 nutrients and each of the four BRIEF-2 and three WASI-II composite scales, accounting for both positive (PWQS) and negative (NWQS) weights to determine the most efficient model. To do so, we first ran unadjusted models with 10 bootstraps and 10 repeated holdouts [23], and no shrinkage parameters (lambda) and the mean Akaike Information Criterion (AIC) was extracted. AIC is a tool widely used for model selection, which highlights the relative quality of different models for a given dataset [24]. Once this was done, three additional models were run, all with 10 bootstraps and 10 repeated holdouts: one model with a lambda 10% of the previously extracted AIC, one model with a lambda 100% of the previously extracted AIC, and lastly a model with a lambda 1000% of the previously extracted AIC. For example, for the BRI BRIEF-2 subscale, the AIC of the initial model was 1500. The lambdas for the four models were 0, 150, 1500, and 15,000, respectively. We then compared the AICs from all four models, and the model with the lowest AIC was chosen for adjusted analyses (Supplemental Table S1). Once the model with the lowest AIC for each BRIEF-2 and WASI-II subscale was identified, we ran covariate-adjusted gWQS models with 100 bootstraps, 100 holdouts, and the lambda selected from the previous step. All models were adjusted for the same set of covariates as the individual nutrient models.

We explored the potential effect modification of these associations by child sex using a product interaction term between child sex and nutrient concentration in individual nutrient analyses. For gWQS analyses, we ran sex-stratified analyses and compared estimates using a test for heterogeneity. These analyses were performed in RStudio using the package “gWQS”.

#### 2.5.2. Single-Nutrient Analysis

As a secondary analysis, we estimated the individual associations between each of the 22 nutrient concentrations with BRIEF-2 and WASI-II index and composite scale T-scores using covariate-adjusted multivariable linear regressions. For the single-nutrient analysis, all nutrients were scaled, and nutrient z-scores were used. All models were adjusted for maternal ethnicity, maternal education, maternal BMI at 26 weeks, maternal age at recruitment, parity, child sex assigned at birth, and child age at assessment.

## 3. Results

### 3.1. Summary Statistics

Of the 1450 mothers enrolled in GUSTO, 1037 were followed until at least 7 years old. Of those mothers, 348 had blood plasma nutrient concentrations and children with BRIEF-2 scores, and 331 had blood plasma nutrient concentrations and children with WASI-II scores. A comparison of the demographics of the entire study population and our analysis population is shown in Supplemental Table S2. Mothers were predominantly Chinese (51%), and the majority had a secondary education or higher (71%) (Table 1). Additionally, mothers had a mean age at recruitment of 30 years and an average mid-pregnancy BMI of 26.8. This was the first pregnancy for 42% of mothers. The children were predominantly male (52%), with a mean age of 7.5 years (Table 1).

Mean maternal blood plasma nutrient concentrations ranged from 0.21 nmol/L (SD: 0.24) for B3-trigonelline to 3297 mg/L (SD: 165) for sodium. Mean nutrient concentrations were comparable when looking at both the BRIEF-2 (348) and WASI-II (311) sample sizes (Table 2). Correlation between nutrients is shown in Supplemental Figure S1, with vitamers of the same parent vitamin being most highly positively correlated, specifically B6 vitamers. Children’s mean BRIEF-2 index, and composite T-scores were 47.9 (SD = 9.8) for the Behavioral Regulation Index, 48.9 (SD: 8.3) for the emotion regulation index, 49.4 (SD = 9.3) for the cognitive regulation index, and 49.6 (SD: 10.0) for the global executive composite score (Table 1). Mean WASI-II block design T-scores were 52.0 (SD: 9.6), 52.9 (SD: 9.7) for Matrix reasoning T-scores, and 104 (SD = 14.6) for Perceptual Reasoning index scores (Table 1).

### 3.2. Nutrient Mixture Analysis

**Figure 1:**
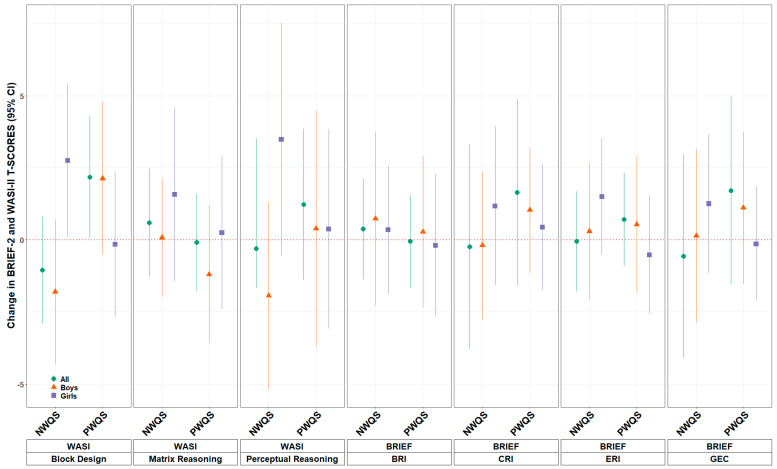
gWQS analysis of the nutrient mixture with BRIEF-2 and WASI-II subscale T-scores in the whole analysis population, in boys only, and in girls only ^a^. ^a^ Models adjusted for maternal ethnicity, maternal education, maternal BMI at 26 weeks, maternal age at recruitment, parity, child sex assigned at birth, and child age at behavioral testing.

**Figure 2:**
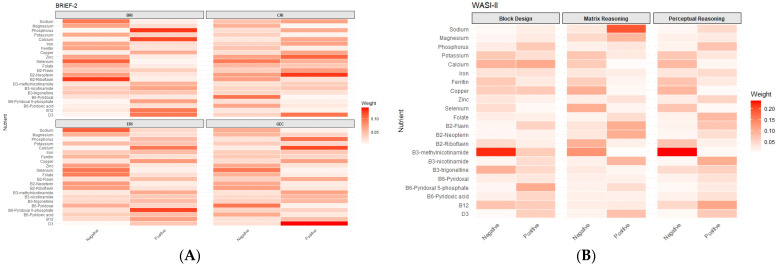
Weights from the gWQS analysis of the 22-nutrient mixture with (**A**) BRIEF-2 and (**B**) WASI-II subscale T-scores.

In gWQS analyses investigating the joint effects of the mixtures, there were no associations found between either positively (BRI β = −0.05 (95% CI: −1.66, 1.55), CRI β = 1.64 (95% CI: −1.60, 4.88), ERI β = 0.70 (95% CI: −0.92, 2.32), and GEC β = 1.71 (95% CI: −1.56, 4.99)) or negatively (BRI β = 0.37 (95% CI: −1.39, 2.12), CRI β = −0.24 (95% CI: −3.79, 3.30), ERI β = −0.06 (95% CI: −1.80, 1.68), and GEC β = −0.57 (95% CI: −4.11, 2.96)) weighted mixtures of nutrients and any BRIEF subscales T-scores (Figure 1, Supplemental Table S3). Maternal exposure to the positive weighted quantile sum of nutrients was associated with higher block design (β = 2.17, 95% CI: 0.03, 4.31) T-scores, as measured by the WASI-II in their children (Figure 1, Supplemental Table S3). This positively weighted mixture was driven by sodium, magnesium, vitamin B3-nicotinamide, vitamin B2-flavin, vitamin B2-neopterin, and vitamin D (Figure 2). There were no other associations with the nutrient mixtures and other WASI-II subscale T-scores.

When examining sex-specific associations, we found a significant difference in regard to the negatively weighted quantile sum mixture and the T-scores from two WASI-II subscales (Figure 1, Supplemental Table S3). Higher concentrations of the negatively weighted quantile sum mixture was associated with lower block design T-scores (β = −1.81 (95% CI: −4.33, 0.71)) and perceptual reasoning (β = −1.94 (95% CI: −5.17, 1.29)) scores in boys and higher block design T-scores (β = 2.75 95% CI: 0.10, 5.40) and null perceptual reasoning (β = 3.48 (95% CI: −0.57, 7.53)) scores in girls (heterogeneity *p*-value = 0.02 and 0.04, respectively).

### 3.3. Individual Nutrient Analysis

**Figure 3:**
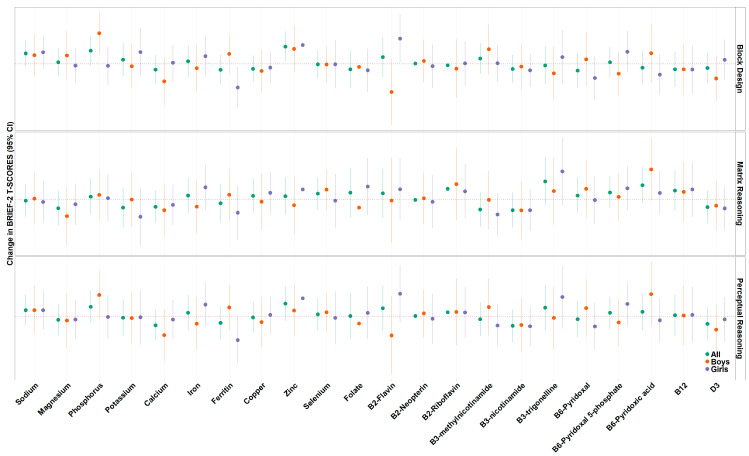
Association between individual maternal nutrient concentrations with BRIEF-2 subscale T-scores in all participants, in boys only, and in girls only. Models have been adjusted for maternal ethnicity, maternal education, maternal BMI at 26 weeks, maternal age at recruitment, parity, child sex assigned at birth, and child age at behavioral testing.

**Figure 4:**
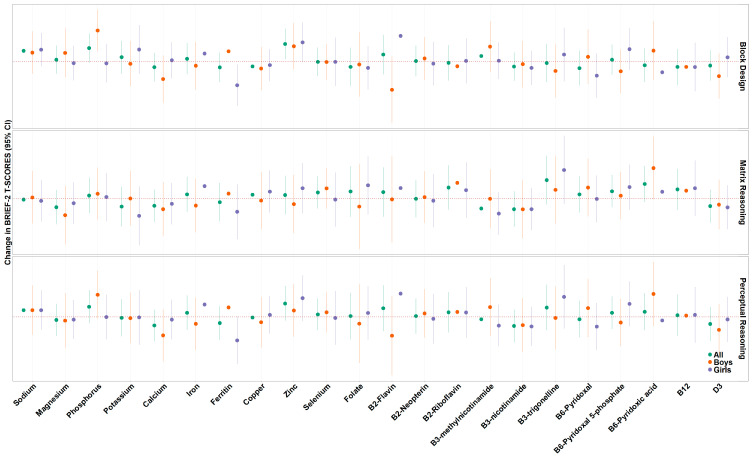
Association between individual maternal nutrient concentrations with WASI-II subscale T-scores in all participants, in boys only, and in girls only. Models have been adjusted for maternal ethnicity, maternal education, maternal BMI at 26 weeks, maternal age at recruitment, parity, child sex assigned at birth, and child age at behavioral testing.

A one standard deviation increase in maternal vitamin B12 concentrations was associated with higher BRI (β = 1.60, 95% CI: 0.10, 3.09), CRI (β = 1.70, 95% CI: 0.28, 3.12), and GEC (β = 1.65, 95% CI: 0.12, 3.18) T-scores as measured by the BRIEF-2. Additionally, a one standard deviation increase in maternal copper concentrations was associated with higher BRI (β = 2.57, 95% CI: 1.07, 4.08), CRI (β = 1.99, 95% CI: 0.61, 3.38), ERI (β = 1.99, 95% CI: 0.59, 3.38), and GEC (β = 2.32, 95% CI: 0.78, 3.85) T-scores (Figure 3, Supplemental Table S5). We found no additional associations between individual nutrients and BRIEF composite scale T-scores measured between 7 and 8 years of age when adjusting for covariates. For WASI-II subscales, a one standard deviation increase in maternal zinc concentrations was associated with higher block design scores (β = 1.8, 95% CI: 0.25, 3.46) (Figure 4, Supplemental Table S6). We found no additional associations between individual nutrients and WASI composite scale T-scores measured between 7 and 8 years of age when adjusting for covariates.

We explored sex-specific associations for each nutrient and BRIEF-2 (Figure 3, Supplemental Table S5) and WASI-II subscale T-scores (Figure 4, Supplemental Table S6). In regard to BRIEF-2 subscales, a one standard deviation increase in maternal phosphorus concentrations was associated with higher BRI (β = 2.49, 95% CI: 0.01, 4.96, heterogeneity *p*-value = 0.04), CRI (β = 2.09, 95% CI: −0.34, 4.52, heterogeneity *p*-value = 0.03), and GEC (β = 2.28, 95% CI: −0.31, 4.88, heterogeneity *p*-value = 0.048) T-scores in boys and null associations in girls. Similarly, we found that a one standard deviation increase in maternal copper concentrations was associated with higher CRI (β = 3.38, 95% CI: 1.44, 5.33, heterogeneity *p*-value = 0.05) and ERI (β = 3.72, 95% CI: 1.78, 5.67, heterogeneity *p*-value = 0.01) T-scores in boys and null associations in girls. A one standard deviation increase in maternal B6-pyridoxal 5-phosphate was associated with higher ERI (β = 2.15, 95% CI: 0.10, 4.20, heterogeneity *p*-value = 0.03) T-scores in boys but null associations in girls. We additionally found that a one standard deviation increase in maternal selenium concentrations were associated with lower BRI (β = −2.70, 95% CI: −4.72, −0.68, heterogeneity *p*-value = 0.03), CRI (β = −2.63, 95% CI: −4.63, −0.64, heterogeneity *p*-value = 0.01), ERI (β = −2.39, 95% CI: −4.10, −0.67, heterogeneity *p*-value = 0.04) and GEC (β = −3.23, 95% CI: −5.33, −1.13, heterogeneity *p*-value = 0.01) T-scores in girls and null associations in boys. In regard to WASI-II subscales, a one-unit increase in phosphorus concentrations was associated with higher block design T-scores (β = 3.28, 95% CI: 1.07, 5.49, heterogeneity *p*-value = 0.02) in boys and null associations in girls. A one-unit increase in ferritin was associated with lower block design T-scores (β = −2.52, 95% CI; −4.71, −0.34, heterogeneity *p*-value 0.02) in girls and a null association in boys. Lastly, a one-unit increase in B2-flavin concentrations was associated with higher block design T-scores (β = 2.72, 95% CI: 0.16, 5.27, heterogeneity *p*-value = 0.01) in girls and marginally significant lower block design T-scores in boys (β = −3.01, −6.55, 0.53).

## 4. Discussion

In this analysis, we investigated the association between maternal essential minerals and vitamins, both individually and as a mixture, with child cognition and executive function T-scores. In our single-nutrient analysis, we found that concentrations of some nutrients, specifically sodium and magnesium, were associated with higher block design T-scores and higher perceptual reasoning index scores as measured in children by the WASI-II. When looking at the entire mixture of nutrients, after adjusting for covariates, the positive weighted quantile sum of nutrients was associated with higher block design T-scores. We did not find associations between individual nutrients or nutrient mixtures and BRIEF-2 scale T-scores.

It has been well documented that nutrition during pregnancy has consequences on offspring cognitive development and function [25]. Pregnant women have higher nutrient requirements, as does the developing fetus [26]. The developmental origins of health and disease theory proposes that maternal nutritional status in the preconception and perinatal periods plays a key role in fetal development. During pregnancy, when the fetus is rapidly developing, the brain has heightened sensitivity to the environment. Several studies have found that maternal obesity, metabolic state, and diet during gestation can all impact the neurodevelopment of the offspring [27]. Not only does maternal nutritional status impact child development, but individual nutrient concentrations have also been shown to be both positively and negatively associated with child executive function.

Increases or decreases of key nutrients during pregnancy can impact brain development and predispose the fetus to postnatal neurodevelopmental disorders [28,29]. The most widely studied nutrient, especially in relation to neurodevelopment, is folate (vitamin B9). Folate was first studied in the 1960s regarding the prevention of neural tube defects [30]. Research has further explored the role of folate in neurodevelopment. There is a large body of literature examining the association between folate and autism, specifically the inverse association between folate concentrations and homocysteine levels and the positive association between increased homocysteine levels and autism [31,32,33]. Although in our study, we did not find any association with folate and either BRIEF-2 or WASI-II scores, this could point to different mechanisms regarding the behaviors measured by these tests. Additionally, other B vitamins have been found to be beneficial regarding offspring behavioral outcomes. A study out of the Kyushu Okinawa Maternal and Child Health Study found that maternal intake of not only folate but vitamins B6 and B2 during pregnancy may be protective against hyperactivity and emotional problems [34]. Similarly, in the West Australian Pregnancy Cohort study, it was found that lower intake of vitamins B1, B2, B3, B5, B6, and folate was associated with higher externalizing behavior scores [35].

Although maternal essential minerals are less widely studied, especially individually, our study found that concentrations of a key essential mineral, magnesium, were positively associated with better visual–spatial ability, motor skills, and problem-solving skills in their children as measured by the block design test. Magnesium has been found to be key to several processes of neurodevelopment such as neuronal activity and neurotransmitter release [36]. A previous study in this cohort found that higher maternal plasma concentrations of magnesium were associated with better child general learning abilities at age 4 [16]. Other minerals are more widely studied, particularly iron, as it has been shown to be important for central nervous system development and functionality. Vaughn et al. first concluded that the mother’s iron-binding capacity was significantly related to irritability in children in early life [37]. However, two studies from Australian cohorts found no association between maternal iron status and child behavior [38,39]. Although sodium is not widely studied in the context of child cognition and executive function, it is known as an important growth factor [40] and is important to cellular function, including that within the brain. There has additionally been research in regard to the deleterious effects of certain nutrients. Copper and zinc have been additionally more widely studied in their association with child behavior. A study of mother–child pairs in Spain found a negative association between maternal copper concentrations and some domains of child neuropsychological development assessed at 12 months and 5 years of age [41]. An additional study found that serum zinc concentrations were correlated with parent-teacher-rated inattention [42]. Sex-specific differences in the association between maternal nutrition and child cognitive outcomes have not been widely studied; however, it suggests different hormonal and nutrient needs in male and female offspring [8]. The sex-specific associations observed in boys likely reflect the heightened vulnerability of male fetuses to prenatal perturbations through several converging mechanisms. First, placental sexual dimorphism plays a critical role: male placentas exhibit distinct gene expression patterns, reduced adaptive capacity to maternal stressors, and lower expression of protective enzymes involved in nutrient transport and oxidative stress defense compared to female placentas, potentially amplifying the consequences of nutrient imbalances [43,44]. Second, hormonal differences during critical windows of brain development may contribute to the sex differences: the male fetal brain undergoes testosterone-driven sexual differentiation during the second trimester, a process requiring precise regulation of zinc-dependent enzymes and magnesium-modulated NMDA receptor signaling, and disruption of these pathways through nutrient deficiencies may disproportionately affect males [45]. Finally, male fetuses exhibit higher baseline metabolic demands and faster growth trajectories, potentially making them more sensitive to micronutrient insufficiencies that compromise mitochondrial function and antioxidant capacity [46,47].

With any study, there are both strengths and limitations. A main limitation of this study is that maternal nutrients were measured from a single sample at one time point during pregnancy. Diet and supplementation are likely to change over the span of pregnancy, so this one window of measurement may not give an accurate picture of the nutrient concentrations the fetus was exposed to and may not capture sensitive windows of development where individual nutrients would be most beneficial or detrimental. Additionally, a limitation of this study is the lack of availability of information regarding child postnatal nutrition or maternal energy intake, preventing adjustment for them in statistical models. A major strength of this study is that we are able to examine a wide range of vitamins, vitamers, and minerals. Not only were we able to explore the effects of these individual nutrients on child cognition and executive function, but we were also able to examine the positive and negative associations of the mixture as a whole. This study expands the current scope of the literature, as the mixture approach regarding nutrients is quite novel. Looking at the mixture of nutrients not only gives a better picture of the nutrients someone consumes but also removes the issue of multiple testing. A weighted index (such as that generated from gWQS) accounts for multicollinearity, identifies key contributing nutrients, and offers a more realistic view of how multiple nutrients interact to affect health. This better reflects real-world scenarios of multiple nutrients with cumulative effects. Additionally, a strength of this study was its ability to look at the association between maternal nutrients and both cognition and executive function. This allowed for a broader understanding of the relationship between maternal nutrition and child neurodevelopment and behavior.

## 5. Conclusions

In this cohort of Singaporean mothers and children, the positive mixture of nutrients, driven by sodium, magnesium, vitamin B3-nicotinamide, vitamin B2-flavin, vitamin B2-neopterin, and vitamin D, was associated with higher block design scores as measured by the WASI-II. This not only supports the current body of literature discussing the importance of maternal nutrition on child health and development but also shines a light on the nutrients that are most positively and negatively associated with child cognition, which help guide further studies. These findings underscore the importance of specific nutrients in prenatal diets.

## Figures and Tables

**Table 1:** GUSTO study participant demographic information.

	N (%)
**Gender**	
Male	182 (52%)
Female	166 (48%)
**Mother Ethnicity**	
Chinese	176 (51%)
Malay	112 (32%)
Indian and Other	60 (17%)
**Mother Education**	
1	20 (5%)
2	82 (24%)
3	129 (38%)
4	113 (33%)
**Parity**	
0	148 (42%)
1	114 (33%)
2 or more	86 (25%)
	**Mean (STD)**
**Maternal Pre-pregnancy BMI**	26.79 (4.66)
**Maternal Age**	30.19 (5.14)
**Child Age**	7.47 (0.18)
**BRIEF-2 Subscales**	
Behavioral Regulation Index (BRI)	47.91 (9.80)
Emotion Regulation Index (ERI)	48.85 (8.31)
Cognitive Regulation Index (CRI)	49.39 (9.31)
Global Executive Composite (GEC)	49.61 (10.0)
**WASI-II Subscales**	
Block Design	52.01 (9.59)
Matrix Reasoning	52.93 (9.71)
Perceptual Reasoning	104.16 (14.59)

**Table 2:** Summary of nutrient concentrations in the BRIEF-2 (N = 348) and WASI-II (N = 331) populations.

Nutrient	BRIEF				WASI			
	Mean	STD	Q25	Q75	Mean	STD	Q25	Q75
**Sodium (mg/L)**	3298	164.79	3216.98	3380.90	3301.92	162.83	3217.31	3383.57
**Magnesium (mg/L)**	19.83	1.83	18.75	20.87	19.86	1.77	18.80	20.84
**Phosphorus (mg/L)**	160.13	21.35	146.55	172.82	161.16	21.69	147.18	174.09
**Potassium (mg/L)**	923.80	163.18	817.05	985.17	931.57	169.03	812.54	1006.24
**Calcium (mg/L)**	89.16	5.90	85.28	92.65	89.19	5.91	85.25	92.63
**Iron (μg/L)**	1080.14	489.89	727.07	1342.54	1108.90	499.57	756.69	1359.61
**Ferritin (ng/mL)**	25.97	10.55	19.39	30.44	26.03	11.24	19.07	30.39
**Copper (μg/L)**	2173.12	418.32	1915.25	2382.63	2142.94	423.83	1894.82	2347.91
**Zinc (μg/L)**	797.52	149.47	713.45	858.76	809.67	157.42	714.41	876.83
**Selenium (μg/L)**	99.65	14.69	89.77	109.07	100.46	15.04	90.07	110.07
**Folate (ng/mL)**	17.91	20.14	9.86	19.17	18.42	20.22	11.00	19.54
**B2-Flavin (nmol/L)**	12.63	9.75	8.56	13.03	12.87	9.91	8.63	13.48
**B2-Neopterin (nmol/L)**	18.33	4.62	15.30	20.70	18.12	4.59	15.00	20.40
**B2-Riboflavin (nmol/L)**	23.61	34.45	9.41	25.65	24.99	35.58	9.87	26.65
**B3-methylnicotinamide (nmol/L)**	262.78	119.92	192.00	313.25	274.19	122.87	197.25	330.50
**B3-nicotinamide (nmol/L)**	194.69	114.22	117.00	253.25	195.50	115.46	117.00	253.00
**B3-trigonelline (μmol/L)**	0.21	0.24	0.09	0.24	0.21	0.23	0.09	0.24
**B6-Pyridoxal (nmol/L)**	27.91	35.67	10.40	33.80	29.05	36.10	10.63	34.18
**B6-Pyridoxal 5-phosphate (nmol/L)**	93.40	69.22	35.78	137.25	99.01	69.45	38.63	146.75
**B6-Pyridoxic acid (nmol/L)**	52.86	58.69	18.08	65.63	55.80	59.39	19.95	70.68
**B12 (pg/mL)**	292.43	106.17	225.95	338.75	301.15	118.33	230.35	349.59
**D3 (nmol/L)**	79.87	27.29	61.00	96.00	84.36	27.09	66.00	101.00

## Data Availability

Data access is governed by applicable local laws and policies and the governance structure of the cohort. Please see https://gustodatavault.sg/about/request-for-data (accessed on 4 February 2026) for details on the procedures for requesting data access.

## Supplementary Materials

The following supporting information can be downloaded at: https://www.mdpi.com/article/10.3390/nu18050818/s1, Table S1: AIC for unadjusted gWQS models at various lambda specifications; Table S2: Demographics in analysis cohort vs. whole study cohort; Table S3: Overall and sex-specific gWQS analysis of the whole nutrient mixture with BRIEF-2 and WASI-II subscale T-scores; Table S4: Weights from the gWQS analysis of the 22 nutrient mixture with BRIEF-2 and WASI-II subscale T-scores; Table S5: Association of individual maternal nutrient concentrations with BRIEF-2 subscale T-scores; Table S6: Overall and sex specific association of individual maternal nutrient concentrations with WASI-II subscale T-scores; Figure S1: Pearson correlation between log 2 transformed maternal nutrient concentrations.

## Abbreviations

The following abbreviations are used in this manuscript:

BRIEF-2	Behavior Rating Inventory of Executive Function 2nd Edition
WASI-II	Wechsler Abbreviated Scale of Intelligence 2nd Edition
gWQS	Generalized Weighted Quantile Sum
