# Supplementary material for: Cognitive and Executive Function Scores at Age 7 in Relation to Maternal Mid-Pregnancy Plasma Nutrient Mixtures in a Singaporean Family Follow-Up Cohort
Source: Nutrients. 2026 Mar 3;18(5):818. doi: 10.3390/nu18050818 (PMC12986960; doi:10.3390/nu18050818)

Supplemental Material

Supplemental Table S1: AIC for unadjusted gWQS models at various lambda specifications

	BRI	CRI	ERI	GEC	Block Design	Matrix Reasoning	Perceptual Reasoning
Lambda 0	1531.8	1506.5	1466.0	1537.4	1430.7	1443.3	1598.6
Lambda 10% AIC	1532.1	1507.2	1466.1	1537.8	1430.4	1443.3	1598.1
Lambda 100% AIC	1532.6	1507.7	1466.4	1538.1	1429.3	1442.3	1597.8
Lambda 1000% AIC	1530.8	1508.4	1466.3	1539.5	1430.6	1439.3	1596.0

Supplemental Table S2: Demographics in analysis cohort vs whole study cohort

	Analysis Cohort N=348)	Whole Cohort N=1089
	N(%)	
**Gender**		
Male	182 (52%)	462 (47%)
Female	166 (48%)	627 (53%)
**Mother Ethnicity**		
Chinese	176 (51%)	616 (56%)
Malay	112 (32%)	277 (26%)
Indian and Other	60 (17%)	196 (18%)
**Mother Education**		
None or Primary School Only	20 (5%)	42 (4%)
Secondary/Technical Education	82 (24%)	392 (36%)
Polytechnical/University Education/Other	242 (71%)	655 (60%)
**Parity**		
Primiparous	148 (42%)	545 (46%)
Multiparous	200 (58%)	643 (54%)
	Mean (STD)	
**Maternal Pre-pregnancy BMI**	26.79 (4.66)	23.70 (4.8)
**Maternal Age**	30.19 (5.14)	30.60 (7.14)

Supplemental Table S3: Overall and sex-specific gWQS analysis of the whole nutrient mixture with BRIEF-2 and WASI-II subscale T-scores

		PWQS				NWQS			
		**All**	**Boys**	**Girls**	**P-value**	**All**	**Boys**	**Girls**	**P-value**
BRIEF-2	**BRI**	-0.05 (-1.66, 1.55)	0.28 (-2.36, 2.91)	-0.20 (-2.68, 2.27)	0.80	0.37 (-1.39, 2.12)	0.73 (-2.29, 3.74)	0.34 (-1.88, 2.57)	0.86
	**CRI**	1.64 (-1.60, 4.88)	1.48 (-3.62, 6.59)	-0.32 (-5.68, 5.04)	0.95	-0.24 (-3.79, 3.30)	-0.28 (-5.59, 5.04)	2.56 (-2.84, 7.95)	0.64
	**ERI**	0.70 (-0.92, 2.32)	1.23 (-3.86, 6.37)	-1.94 (-7.06, 3.18)	0.50	-0.06 (-1.80, 1.68)	-0.53 (-5.41, 4.35)	3.01 (-2.05, 8.07)	0.45
	**GEC**	1.71 (-1.56, 4.99)	1.78 (-4.10, 7.67)	-1.15 (-6.59, 4.29)	0.46	-0.57 (-4.11, 2.96)	-0.34 (-6.24, 5.56)	2.76 (-2.56, 8.08)	0.57
WASI-II	**Block Design**	2.17 (0.03, 4.31)	2.39 (-0.56, 5.34)	-0.24 (-3.40, 2.92)	0.22	-1.06 (-2.92, 0.79)	-2.25 (-4.92, 0.41)	2.83 (-0.46, 6.12)	0.02
	**Matrix Reasoning**	-0.09 (-1.78, 1.59)	-1.21 (-3.60, 1.19)	0.25 (-2.41, 2.90)	0.42	0.59 (-1.28, 2.45)	0.08 (-1.98, 2.13)	1.57 (-1.45, 4.60)	0.42
	**Perceptual Reasoning**	1.22 (-1.40, 3.85)	0.39 (-3.72, 4.49)	0.37 (-3.09, 3.84)	0.99	-0.31 (-1.68, 3.52)	-1.94 (-5.17, 1.29)	3.48 (-0.57, 7.53)	0.04

Supplemental Table S4: Weights from the gWQS analysis of the 22 nutrient mixture with BRIEF-2 and WASI-II subscale T-scores

Nutrients	BRIEF-2								WASI-II					
	BRI		CRI		ERI		GEC		Block Design		Perceptual Reasoning		Matrix Reasoning	
	Positive	Negative	Positive	Negative	Positive	Negative	Positive	Negative	Positive	Negative	Positive	Negative	Positive	Negative
**Sodium**	0.012	0.096	0.029	0.045	0.011	0.112	0.021	0.062	0.192	0.008	0.047	0.030	0.124	0.010
**Magnesium**	0.025	0.067	0.024	0.058	0.030	0.057	0.026	0.054	0.09	0.011	0.029	0.048	0.060	0.019
**Phosphorus**	0.129	0.006	0.050	0.025	0.100	0.009	0.066	0.022	0.032	0.025	0.074	0.018	0.047	0.015
**Potassium**	0.033	0.063	0.044	0.036	0.030	0.050	0.041	0.038	0.028	0.068	0.031	0.072	0.029	0.070
**Calcium**	0.121	0.009	0.096	0.018	0.122	0.008	0.106	0.016	0.005	0.095	0.013	0.064	0.005	0.087
**Iron**	0.026	0.064	0.050	0.034	0.041	0.038	0.040	0.037	0.023	0.035	0.045	0.031	0.033	0.034
**Ferritin**	0.022	0.068	0.024	0.045	0.032	0.054	0.027	0.040	0.014	0.067	0.027	0.062	0.019	0.075
**Copper**	0.059	0.013	0.073	0.025	0.069	0.019	0.066	0.026	0.009	0.062	0.007	0.100	0.007	0.088
**Zinc**	0.014	0.066	0.017	0.067	0.020	0.071	0.018	0.063	0.037	0.033	0.054	0.023	0.044	0.025
**Selenium**	0.008	0.109	0.011	0.094	0.009	0.097	0.009	0.096	0.014	0.043	0.009	0.101	0.009	0.071
**Folate**	0.015	0.058	0.024	0.052	0.015	0.093	0.022	0.057	0.043	0.023	0.086	0.015	0.067	0.016
**B2-Flavin**	0.036	0.041	0.048	0.036	0.061	0.023	0.054	0.034	0.103	0.011	0.071	0.035	0.100	0.015
**B2-Neopterin**	0.019	0.072	0.015	0.070	0.017	0.074	0.016	0.064	0.101	0.011	0.042	0.031	0.078	0.012
**B2-Riboflavin**	0.013	0.128	0.022	0.069	0.019	0.071	0.021	0.070	0.019	0.045	0.019	0.098	0.012	0.073
**B3-methylnicotinamide**	0.056	0.023	0.062	0.023	0.067	0.018	0.063	0.023	0.003	0.221	0.008	0.132	0.002	0.236
**B3-nicotinamide**	0.067	0.036	0.039	0.031	0.054	0.029	0.043	0.029	0.088	0.013	0.102	0.018	0.104	0.010
**B3-trigonelline**	0.042	0.041	0.050	0.031	0.052	0.029	0.048	0.033	0.013	0.097	0.058	0.028	0.030	0.046
**B6-Pyridoxal**	0.017	0.023	0.032	0.099	0.010	0.059	0.032	0.097	0.019	0.024	0.037	0.02	0.029	0.017
**B6-Pyridoxal 5-phosphate**	0.069	0.007	0.124	0.024	0.033	0.023	0.104	0.031	0.042	0.011	0.027	0.017	0.034	0.009
**B6-Pyridoxic acid**	0.024	0.013	0.049	0.066	0.018	0.037	0.051	0.063	0.018	0.018	0.038	0.019	0.023	0.016
**B12**	0.096	0.018	0.069	0.019	0.048	0.028	0.063	0.022	0.026	0.073	0.109	0.023	0.056	0.048
**D3**	0.099	0.009	0.042	0.032	0.141	0.004	0.061	0.023	0.079	0.007	0.066	0.015	0.087	0.008

Supplemental Table S5: Association of individual maternal nutrient concentrations with BRIEF-2 subscale T-scores[a]

Nutrient	BRI				CRI				ERI				GEC			
	All	Boys	Girls	P-value*	All	Boys	Girls	P-value*	All	Boys	Girls	P-value*	All	Boys	Girls	P-value*
**Sodium**	0.46 (-1.04, 1.95)	0.81 (-1.23, 2.84)	0.04 (-2.16, 2.25)	0.62	0.17 (-1.25, 1.58)	0.76 (-1.16, 2.69)	-0.54 (-2.62, 1.55)	0.37	-0.15 (-1.45, 1.15)	-0.16 (-1.92, 1.61)	-0.14 (-2.06, 1.77)	0.99	0.16 (-1.4, 1.71)	0.6 (-1.51, 2.71)	-0.36 (-2.65, 1.93)	0.55
**Magnesium**	0.48 (-1.15, 2.12)	0.37 (-2.13, 2.87)	0.57 (-1.6, 2.74)	0.90	0.22 (-1.33, 1.76)	0.84 (-1.51, 3.19)	-0.25 (-2.29, 1.79)	0.49	-0.25 (-1.65, 1.15)	-0.28 (-2.42, 1.86)	-0.22 (-2.08, 1.63)	0.97	0.14 (-1.55, 1.82)	0.43 (-2.14, 3.01)	-0.09 (-2.32, 2.14)	0.76
**Phosphorus**	0.52 (-1.07, 2.11)	2.49 (0.01, 4.96)	-0.81 (-2.85, 1.22)	0.04	-0.03 (-1.59, 1.53)	2.09 (-0.34, 4.52)	-1.46 (-3.46, 0.53)	0.03	0.71 (-0.67, 2.1)	1.91 (-0.27, 4.08)	-0.09 (-1.88, 1.69)	0.16	0.26 (-1.41, 1.92)	2.28 (-0.31, 4.88)	-1.11 (-3.25, 1.02)	0.05
**Potassium**	0.96 (-0.88, 2.81)	0.47 (-2.41, 3.35)	1.32 (-1.12, 3.75)	0.66	0.51 (-1.26, 2.27)	0.64 (-2.12, 3.41)	0.41 (-1.93, 2.75)	0.90	0.94 (-0.58, 2.45)	1.22 (-1.16, 3.6)	0.73 (-1.28, 2.74)	0.76	0.74 (-1.15, 2.63)	0.74 (-2.22, 3.69)	0.74 (-1.75, 3.24)	1.00
**Calcium**	0.23 (-1.3, 1.76)	-0.02 (-2.19, 2.14)	0.48 (-1.7, 2.67)	0.75	0.09 (-1.37, 1.56)	-0.24 (-2.32, 1.84)	0.43 (-1.66, 2.52)	0.66	0.12 (-1.22, 1.45)	0.45 (-1.45, 2.34)	-0.21 (-2.12, 1.69)	0.63	0.19 (-1.39, 1.77)	0.03 (-2.22, 2.27)	0.36 (-1.9, 2.62)	0.84
**Iron**	-0.17 (-1.89, 1.56)	0.44 (-1.94, 2.82)	-0.77 (-3.14, 1.6)	0.48	0.31 (-1.34, 1.96)	0.95 (-1.32, 3.23)	-0.33 (-2.59, 1.94)	0.44	-0.79 (-2.24, 0.66)	-0.26 (-2.26, 1.74)	-1.31 (-3.3, 0.69)	0.47	-0.2 (-1.98, 1.58)	0.49 (-1.96, 2.94)	-0.88 (-3.32, 1.56)	0.44
**Ferritin**	1.17 (-0.52, 2.86)	1.02 (-1.53, 3.56)	1.29 (-0.96, 3.53)	0.88	1.28 (-0.35, 2.91)	2.47 (0.03, 4.91)	0.36 (-1.79, 2.51)	0.20	0.59 (-0.81, 1.99)	0.72 (-1.4, 2.84)	0.49 (-1.38, 2.36)	0.87	1.27 (-0.46, 3)	1.93 (-0.68, 4.54)	0.76 (-1.54, 3.06)	0.51
**Copper**	2.57 (1.07, 4.08)	3.65 (1.51, 5.78)	1.56 (-0.51, 3.63)	0.17	1.99 (0.61, 3.38)	3.38 (1.44, 5.33)	0.68 (-1.21, 2.57)	0.05	1.99 (0.59, 3.38)	3.72 (1.78, 5.67)	0.35 (-1.53, 2.24)	0.01	2.32 (0.78, 3.85)	3.81 (1.65, 5.98)	0.91 (-1.19, 3.01)	0.06
**Zinc**	0.37 (-1.33, 2.07)	0.68 (-1.81, 3.18)	0.1 (-2.2, 2.41)	0.74	0.35 (-1.23, 1.93)	1.41 (-0.9, 3.72)	-0.55 (-2.68, 1.59)	0.22	0.58 (-0.87, 2.03)	0.57 (-1.56, 2.7)	0.59 (-1.37, 2.55)	0.99	0.43 (-1.27, 2.13)	1.07 (-1.43, 3.57)	-0.11 (-2.42, 2.2)	0.50
**Selenium**	-1.29 (-2.83, 0.25)	0.69 (-1.72, 3.09)	-2.7 (-4.72, -0.68)	0.03	-1.01 (-2.54, 0.52)	1.28 (-1.1, 3.65)	-2.63 (-4.63, -0.64)	0.01	-1.21 (-2.51, 0.1)	0.45 (-1.58, 2.49)	-2.39 (-4.1, -0.67)	0.04	-1.48 (-3.09, 0.14)	0.99 (-1.51, 3.49)	-3.23 (-5.33, -1.13)	0.01
**Folate**	-1.51 (-3.40, 0.38)	-0.5 (-4.83, 3.84)	-1.72 (-3.79, 0.35)	0.62	-0.79 (-2.62, 1.03)	2.18 (-1.94, 6.3)	-1.42 (-3.39, 0.55)	0.12	-0.74 (-2.38, 0.9)	0.37 (-3.39, 4.13)	-0.97 (-2.77, 0.82)	0.53	-1.16 (-3.12, 0.8)	1.19 (-3.27, 5.65)	-1.66 (-3.79, 0.47)	0.26
**B2-Flavin**	0.79 (-1.19, 2.77)	-0.3 (-3.48, 2.88)	1.58 (-1.1, 4.25)	0.38	0.94 (-1.05, 2.93)	-0.05 (-3.25, 3.14)	1.66 (-1.02, 4.34)	0.42	1.02 (-0.7, 2.74)	-0.13 (-2.89, 2.63)	1.85 (-0.47, 4.16)	0.28	1.15 (-0.98, 3.27)	-0.12 (-3.52, 3.28)	2.06 (-0.8, 4.92)	0.34
**B2-Neopterin**	-0.04 (-1.60, 1.51)	0.79 (-1.43, 3.01)	-0.87 (-3.08, 1.34)	0.30	-0.16 (-1.65, 1.34)	0.75 (-1.37, 2.88)	-1.06 (-3.18, 1.06)	0.24	-0.28 (-1.63, 1.07)	0.46 (-1.47, 2.39)	-1.01 (-2.94, 0.91)	0.29	-0.22 (-1.85, 1.41)	0.73 (-1.59, 3.05)	-1.17 (-3.49, 1.14)	0.25
**B2-Riboflavin**	1.47 (-0.37, 3.30)	1.76 (-0.63, 4.15)	0.98 (-2.16, 4.13)	0.70	0.51 (-1.26, 2.29)	0.63 (-1.69, 2.95)	0.32 (-2.73, 3.37)	0.87	1.45 (-0.27, 3.17)	1.68 (-0.57, 3.92)	1.08 (-1.87, 4.03)	0.75	1.06 (-0.87, 3)	1.19 (-1.33, 3.71)	0.85 (-2.47, 4.17)	0.87
**B3-methylnicotinamide**	-1.55 (-3.07, -0.04)	-1.98 (-4.47, 0.5)	-1.3 (-3.22, 0.62)	0.67	-0.29 (-1.73, 1.16)	-1.73 (-4.09, 0.63)	0.58 (-1.25, 2.4)	0.13	-0.79 (-2.1, 0.52)	-0.89 (-3.04, 1.26)	-0.74 (-2.4, 0.93)	0.91	-0.74 (-2.29, 0.81)	-1.76 (-4.3, 0.77)	-0.13 (-2.08, 1.83)	0.32
**B3-nicotinamide**	0.22 (-1.45, 1.89)	1.98 (-0.69, 4.65)	-0.84 (-2.92, 1.25)	0.10	0.79 (-0.85, 2.42)	1.53 (-1.09, 4.16)	0.34 (-1.72, 2.39)	0.48	0.19 (-1.26, 1.65)	1.68 (-0.65, 4)	-0.7 (-2.52, 1.11)	0.11	0.54 (-1.19, 2.28)	1.71 (-1.08, 4.5)	-0.16 (-2.34, 2.02)	0.30

**B3-trigonelline**	-1.68 (-3.80, 0.43)	-2.96 (-5.69, -0.24)	-0.05 (-3.1, 3.01)	0.16	-1.24 (-3.23, 0.75)	-1.96 (-4.55, 0.62)	-0.32 (-3.22, 2.58)	0.41	-1.41 (-3.34, 0.51)	-2.69 (-5.16, -0.22)	0.22 (-2.55, 3)	0.12	-1.43 (-3.62, 0.75)	-2.41 (-5.24, 0.41)	-0.17 (-3.35, 3)	0.30
**B6-Pyridoxal**	-0.1 (-1.94, 1.75)	-0.18 (-2.9, 2.53)	-0.02 (-2.49, 2.44)	0.93	0.86 (-0.91, 2.63)	1.41 (-1.19, 4.02)	0.4 (-1.97, 2.78)	0.57	0.61 (-1.04, 2.27)	1.29 (-1.14, 3.72)	0.05 (-2.16, 2.26)	0.46	0.76 (-1.17, 2.69)	1.16 (-1.67, 4)	0.42 (-2.16, 3.01)	0.71
**B6-Pyridoxal 5-phosphate**	0.37 (-1.26, 2.00)	1.37 (-0.93, 3.67)	-0.61 (-2.88, 1.66)	0.23	0.24 (-1.36, 1.84)	1.54 (-0.71, 3.78)	-1.03 (-3.25, 1.19)	0.11	0.5 (-0.97, 1.97)	2.15 (0.1, 4.2)	-1.12 (-3.14, 0.91)	0.03	0.33 (-1.4, 2.06)	1.78 (-0.64, 4.21)	-1.1 (-3.5, 1.3)	0.10
**B6-Pyridoxic acid**	-0.14 (-1.92, 1.64)	0.43 (-2.45, 3.3)	-0.49 (-2.75, 1.77)	0.62	0.26 (-1.46, 1.97)	0.62 (-2.15, 3.4)	0.03 (-2.15, 2.21)	0.74	-0.15 (-1.73, 1.42)	0.47 (-2.08, 3.01)	-0.54 (-2.54, 1.47)	0.54	0.06 (-1.79, 1.91)	0.54 (-2.45, 3.53)	-0.23 (-2.58, 2.12)	0.69
**B12**	1.6 (0.10, 3.09)	1.47 (-0.45, 3.4)	1.78 (-0.6, 4.17)	0.84	1.7 (0.28, 3.12)	1.1 (-0.72, 2.92)	2.63 (0.37, 4.88)	0.30	0.75 (-0.59, 2.09)	-0.02 (-1.72, 1.69)	1.93 (-0.18, 4.05)	0.16	1.65 (0.12, 3.18)	0.92 (-1.03, 2.88)	2.79 (0.36, 5.21)	0.24
**D3**	0.12 (-1.44, 1.68)	-0.11 (-2.19, 1.96)	0.39 (-1.86, 2.65)	0.75	-0.22 (-1.72, 1.28)	-0.22 (-2.22, 1.78)	-0.22 (-2.39, 1.95)	1.00	0.04 (-1.35, 1.44)	0.4 (-1.46, 2.25)	-0.38 (-2.39, 1.63)	0.58	-0.08 (-1.71, 1.56)	-0.01 (-2.19, 2.16)	-0.15 (-2.51, 2.21)	0.93

[a] Models adjusted for maternal ethnicity, maternal education, maternal BMI at 26 weeks, maternal age at recruitment, parity, child sex-assigned at birth, child age at behavioral testing.

Supplemental Table S6: Overall and sex specific association of individual maternal nutrient concentrations with WASI-II subscale T-scores[a]

	Block Design				Matrix Reasoning				Perceptual Reasoning			
	**All**	**Boys**	**Girls**	**P-Value***	**All**	**Boys**	**Girls**	**P-Value***	**All**	**Boys**	**Girls**	**P-Value***
**Sodium**	1.13 (-0.27, 2.52)	0.93 (-1.32, 3.18)	1.25 (-0.53, 3.03)	0.83	-0.11 (-1.52, 1.29)	0.07 (-2.19, 2.33)	-0.23 (-2.02, 1.56)	0.84	0.9 (-1.18, 2.98)	0.89 (-2.46, 4.24)	0.9 (-1.75, 3.55)	1.00
**Magnesium**	0.18 (-1.33, 1.68)	0.91 (-1.67, 3.48)	-0.19 (-2.03, 1.65)	0.50	-0.78 (-2.25, 0.7)	-1.47 (-4, 1.06)	-0.43 (-2.23, 1.37)	0.51	-0.43 (-2.58, 1.73)	-0.52 (-4.22, 3.19)	-0.38 (-3.02, 2.26)	0.93
**Phosphorus**	1.41 (-0.13, 2.96)	3.28 (1.07, 5.49)	-0.2 (-2.26, 1.86)	0.02	0.24 (-1.29, 1.77)	0.39 (-1.85, 2.63)	0.1 (-1.98, 2.19)	0.85	1.36 (-0.87, 3.6)	2.98 (-0.26, 6.23)	-0.04 (-3.06, 2.98)	0.74
**Potassium**	0.45 (-1.3, 2.2)	-0.25 (-2.64, 2.14)	1.27 (-1.32, 3.85)	0.40	-0.71 (-2.44, 1.01)	-0.02 (-2.38, 2.33)	-1.52 (-4.07, 1.02)	0.40	-0.14 (-2.64, 2.37)	-0.19 (-3.62, 3.24)	-0.08 (-3.79, 3.63)	0.95
**Calcium**	-0.62 (-2.14, 0.9)	-1.88 (-4.39, 0.63)	0.12 (-1.8, 2.04)	0.22	-0.65 (-2.05, 0.75)	-0.94 (-3.26, 1.38)	-0.48 (-2.25, 1.3)	0.76	-1.17 (-3.29, 0.95)	-2.52 (-6.03, 0.99)	-0.37 (-3.06, 2.31)	0.75
**Iron**	0.29 (-1.36, 1.94)	-0.45 (-2.97, 2.07)	0.83 (-1.34, 3)	0.45	0.33 (-1.19, 1.85)	-0.64 (-2.95, 1.68)	1.04 (-0.95, 3.03)	0.28	0.55 (-1.79, 2.89)	-0.95 (-4.52, 2.62)	1.66 (-1.41, 4.73)	0.68
**Ferritin**	-0.63 (-2.19, 0.93)	1.08 (-1, 3.16)	-2.52 (-4.71, -0.34)	0.02	-0.34 (-1.99, 1.31)	0.41 (-1.83, 2.65)	-1.18 (-3.53, 1.18)	0.34	-0.86 (-3.13, 1.42)	1.26 (-1.79, 4.31)	-3.2 (-6.41, 0.01)	0.66
**Copper**	-0.52 (-1.91, 0.86)	-0.76 (-3.06, 1.54)	-0.4 (-2.1, 1.3)	0.80	0.3 (-1.17, 1.78)	-0.2 (-2.65, 2.25)	0.57 (-1.24, 2.39)	0.62	-0.09 (-2.16, 1.97)	-0.74 (-4.17, 2.69)	0.26 (-2.28, 2.79)	0.74
**Zinc**	1.85 (0.25, 3.46)	1.61 (-0.82, 4.05)	2.03 (-0.09, 4.15)	0.80	0.27 (-1.38, 1.92)	-0.5 (-3, 2)	0.86 (-1.32, 3.04)	0.42	1.8 (-0.54, 4.15)	0.86 (-2.7, 4.42)	2.51 (-0.58, 5.61)	0.56
**Selenium**	-0.05 (-1.57, 1.46)	-0.06 (-1.97, 1.85)	-0.04 (-2.61, 2.53)	0.99	0.5 (-0.89, 1.89)	0.85 (-0.9, 2.6)	-0.12 (-2.47, 2.22)	0.52	0.33 (-1.82, 2.48)	0.6 (-2.11, 3.32)	-0.16 (-3.8, 3.49)	0.79
**Folate**	-0.58 (-2.6, 1.44)	-0.32 (-3.74, 3.09)	-0.68 (-3, 1.64)	0.87	0.58 (-1.61, 2.76)	-0.73 (-4.4, 2.94)	1.11 (-1.38, 3.6)	0.42	0.09 (-3.07, 3.26)	-0.94 (-6.26, 4.39)	0.51 (-3.11, 4.13)	0.73
**B2-Flavin**	0.72 (-1.38, 2.83)	-3.01 (-6.55, 0.53)	2.72 (0.16, 5.27)	0.01	0.53 (-1.6, 2.66)	-0.11 (-3.82, 3.61)	0.87 (-1.82, 3.56)	0.68	1.16 (-1.96, 4.27)	-2.57 (-7.92, 2.78)	3.14 (-0.73, 7.01)	0.68
**B2-Neopterin**	0.04 (-1.59, 1.68)	0.31 (-1.95, 2.58)	-0.24 (-2.56, 2.07)	0.74	-0.05 (-1.67, 1.57)	0.1 (-2.15, 2.34)	-0.21 (-2.5, 2.08)	0.85	0.09 (-2.29, 2.48)	0.45 (-2.86, 3.75)	-0.28 (-3.66, 3.09)	0.77
**B2-Riboflavin**	-0.15 (-2.04, 1.74)	-0.51 (-3.65, 2.63)	0.05 (-2.31, 2.42)	0.78	0.92 (-0.99, 2.83)	1.33 (-1.84, 4.5)	0.69 (-1.7, 3.09)	0.75	0.61 (-2.13, 3.34)	0.66 (-3.89, 5.2)	0.58 (-2.85, 4.01)	0.97
**B3-methylnicotinamide**	0.58 (-1.01, 2.18)	1.57 (-1.12, 4.27)	0.07 (-1.88, 2.02)	0.38	-0.89 (-2.4, 0.61)	-0.05 (-2.6, 2.49)	-1.33 (-3.17, 0.52)	0.43	-0.34 (-2.66, 1.98)	1.31 (-2.59, 5.22)	-1.19 (-4.02, 1.64)	0.69
**B3-nicotinamide**	-0.55 (-2.06, 0.95)	-0.29 (-2.76, 2.18)	-0.69 (-2.49, 1.12)	0.80	-0.94 (-2.47, 0.58)	-0.95 (-3.46, 1.55)	-0.94 (-2.77, 0.89)	0.99	-1.24 (-3.46, 0.97)	-1.13 (-4.76, 2.51)	-1.3 (-3.96, 1.36)	0.91
**B3-trigonelline**	-0.16 (-2.19, 1.87)	-1.01 (-3.82, 1.79)	0.72 (-2.13, 3.57)	0.40	1.56 (-0.54, 3.67)	0.72 (-2.19, 3.63)	2.44 (-0.51, 5.39)	0.42	1.25 (-1.82, 4.32)	-0.16 (-4.41, 4.09)	2.7 (-1.61, 7.01)	0.64
**B6-Pyridoxal**	-0.71 (-2.55, 1.12)	0.49 (-2.41, 3.39)	-1.51 (-3.87, 0.85)	0.30	0.33 (-1.24, 1.89)	0.92 (-1.56, 3.4)	-0.07 (-2.09, 1.95)	0.55	-0.33 (-2.8, 2.14)	1.18 (-2.74, 5.1)	-1.33 (-4.52, 1.86)	0.69
**B6-Pyridoxal 5-phosphate**	0.18 (-1.42, 1.79)	-1.05 (-3.39, 1.28)	1.3 (-0.92, 3.52)	0.15	0.6 (-0.79, 2)	0.21 (-1.83, 2.26)	0.96 (-0.99, 2.9)	0.60	0.54 (-1.65, 2.74)	-0.79 (-3.99, 2.42)	1.75 (-1.3, 4.8)	0.67
**B6-Pyridoxic acid**	-0.41 (-2.2, 1.39)	1.15 (-1.96, 4.25)	-1.15 (-3.32, 1.02)	0.23	1.22 (-0.34, 2.78)	2.6 (-0.1, 5.3)	0.56 (-1.33, 2.44)	0.22	0.67 (-1.84, 3.18)	3.09 (-1.26, 7.44)	-0.49 (-3.53, 2.54)	0.73
**B12**	-0.59 (-2.5, 1.32)	-0.57 (-3.36, 2.21)	-0.6 (-3.19, 1.99)	0.99	0.77 (-1.01, 2.54)	0.65 (-1.94, 3.23)	0.87 (-1.53, 3.27)	0.90	0.21 (-2.59, 3)	0.16 (-3.91, 4.24)	0.25 (-3.54, 4.03)	0.97
**D3**	-0.44 (-2.04, 1.16)	-1.57 (-3.97, 0.83)	0.45 (-1.68, 2.57)	0.22	-0.68 (-2.09, 0.73)	-0.55 (-2.68, 1.58)	-0.78 (-2.68, 1.11)	0.87	-0.98 (-3.27, 1.31)	-1.76 (-5.21, 1.69)	-0.36 (-3.43, 2.7)	0.77

[a] Models adjusted for maternal ethnicity, maternal education, maternal BMI at 26 weeks, maternal age at recruitment, parity, child sex-assigned at birth, child age at behavioral testing.

*P-value for test of heterogeneity between estimates from analysis with boys only compared to girls only

Supplemental Figure S1: Pearson correlation between log 2 transformed maternal nutrient concentrations

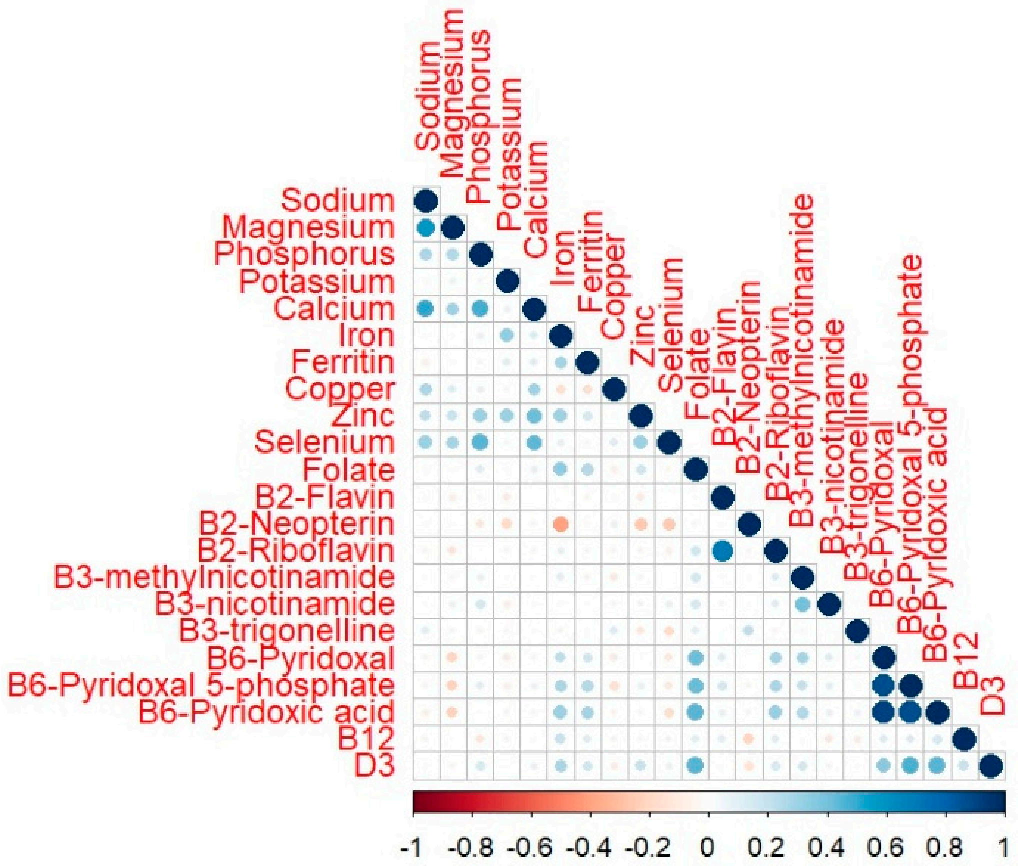

Supplement: Supplementary file 1 [file nutrients-18-00818-s001.zip › nutrients-4125374-supplementary.pdf]
